# Erythropoiesis in Cushing syndrome: sex-related and subtype-specific differences. Results from a monocentric study

**DOI:** 10.1007/s40618-023-02128-x

**Published:** 2023-06-14

**Authors:** M. Detomas, T. Deutschbein, M. Tamburello, I. Chifu, O. Kimpel, S. Sbiera, M. Kroiss, M. Fassnacht, B. Altieri

**Affiliations:** 1 Department of Internal Medicine I, Division of Endocrinology and Diabetes, University Hospital Würzburg, University of Würzburg, Oberdürrbacher Straße 6, 97080 Würzburg, Germany; 2 Medicover Oldenburg MVZ, Oldenburg, Germany; 3 Section of Pharmacology, Department of Molecular and Translational Medicine, University of Brescia, 25124 Brescia, Italy; 4 Department of Internal Medicine IV, University Hospital Munich, Ludwig-Maximilians-Universität München, Munich, Germany; 5 Central Laboratory, University Hospital Würzburg, Würzburg, Germany

**Keywords:** Anemia, Glucocorticoid excess, Hemoglobin, Hypercortisolism, Hypogonadisms, Major cardiovascular event, Menstrual cycle, Red blood cells, Sex

## Abstract

**Context:**

Cushing syndrome (CS) is associated with different hematological abnormalities. Nevertheless, conflicting data about erythropoiesis in CS have been reported. Furthermore, it is unclear whether CS sex and subtype-specific alterations in red blood cells (RBC) parameters are present.

**Objective:**

To investigate sex and subtype-specific changes in RBC in patients with CS at initial diagnosis and after remission.

**Design:**

Retrospective, monocentric study including 210 patients with CS (women, n = 162) matched 1:1 for sex and age to patients with pituitary microadenomas or adrenal incidentalomas (both hormonally inactive). RBC parameters were evaluated at initial diagnosis and after remission.

**Results:**

Women with CS had higher hematocrit (median 42.2 *vs* 39.7%), hemoglobin (14.1 *vs* 13.4 g/dl) and mean corpuscular volume (MCV) (91.2 *vs* 87.9 fl) compared to the controls (all *p* < 0.0001). Women with Cushing disease (CD) showed higher hematocrit, RBC and hemoglobin levels than those with ectopic Cushing (ECS) (all *p* < 0.005). Men with CS had lower hematocrit (42.9 *vs* 44.7%), RBC count (4.8 *vs* 5.1n*10^6^/µl) and hemoglobin (14.2 *vs* 15.4 g/dl), but higher MCV (90.8 *vs* 87.5 fl) than controls (all *p* < 0.05). In men with CS, no subtype-specific differences were identified. Three months after remission hemoglobin decreased in both sexes.

**Conclusion:**

CS is characterized by sexual and subtype-specific differences in RBC parameters. Compared to controls, women with CS showed higher hematocrit/hemoglobin levels, whereas men had lower hematocrit/hemoglobin, which further decreased directly after remission. Therefore, anemia should be considered as complication of CS in men. In women, differences in RBC parameters may help to differentiate CD from ECS.

**Supplementary Information:**

The online version contains supplementary material available at 10.1007/s40618-023-02128-x.

## Introduction

Endogenous Cushing syndrome (CS) is a rare condition resulting from an adrenocorticotropin (ACTH)-dependent or ACTH-independent hypercortisolemia. Apart from well-known clinical features such as hypertension, osteoporosis, and muscle atrophy [1], glucocorticoid excess is associated to hematological alterations like neutrophilia, lymphopenia and eosinopenia [2].

Although it is known that glucocorticoids play in vitro and in vivo an important role in stimulating erythropoiesis [3–5] and that patients with adrenal insufficiency are characterized by normocytic normochromic anemia [6], only few studies on erythropoiesis in CS patients have been performed [5, 7]. In a recent study, high numbers of erythrocytes were identified in patients with overt CS [5], and in two reports polycythemia has been described as one of the first manifestations of CS [8, 9]. On the contrary, an Italian study evaluating a cohort of 80 patients with active Cushing disease (CD), reported anemia and low numbers of red blood cells (RBC) in men, while women did not show any relevant alterations [7].

For these reasons, it is currently unclear whether and what alterations in RBC parameters develop in a sex-dependent manner. Furthermore, since the studies on erythropoiesis and CS have been performed on CD patients, it remains unknown if there are some CS subtype specific differences. Finally, it is not clear if and eventually how fast the RBC parameters normalize after recovery from CS.

The aim of our study was to investigate RBC parameters in a large cohort of patients with CS, evaluating potential subtype-specific and sex-dependent discrepancies, in comparison with matched patients diagnosed with a hormonally inactive pituitary microadenomas (without any pituitary insufficiency) or hormonally inactive adrenal incidentalomas. Furthermore, the reversibility of RBC changes after surgical remission were evaluated during follow-up at different time points.

## Subjects and methods

### Study design and population

A retrospective study in patients with endogenous CS who were treated at the Division of Endocrinology and Diabetes of the University Hospital Würzburg between January 2000 and June 2022 was performed. Patients were identified via chart review and matched 1:1 according to age and sex to patients with hormonally inactive pituitary microadenomas (without pituitary insufficiency) or endocrine inactive adrenocortical incidentalomas, who served as control group. Diagnosis of ACTH-dependent CS (including Cushing disease, CD and ectopic CS, ECS) and ACTH-independent CS (due to cortisol-producing adrenocortical adenomas, CPA and carcinomas, ACC) was made according to established diagnostic criteria [10, 11]. In the control group, cortisol hypersecretion was excluded following the diagnostic algorithm suggested by the current guidelines [11, 12]. Particularly, in patients with adrenal incidentaloma also mild autonomous cortisol secretion was excluded (e.g. 1 mg dexamethasone suppression test, DST, below 1.8 µg/dl, urinary free cortisol between 8 and 70 µg/24 h, and late night salivary cortisol between 0 and 0.15 µg/dl) [12].

All patients with RBC count parameters at the time of the initial diagnosis of CS were considered eligible. Patients were excluded if one of the following conditions was present in a time interval of 4 weeks before blood sampling: (i) supplementation with iron, vitamin B12, and/or folate, (ii) piles or gastric ulcera, (iii) overt hypothyroidism, (iv) hematological or malignant diseases (except if they were causative for the CS), (v) renal disease, and (vi) administration of chemotherapy.

The analysis of RBC parameters in CS patients included 4 time points: at initial diagnosis and during the follow-up at 3, 12, and ≥ 24 months after biochemical remission (only considered as surgical removal of the causative tumor). At time of diagnosis, patients were also stratified based on the duration of the disease, considered as the time between the beginning of symptoms typical of CS (including hypertension, hyperglycemia, muscle atrophy, weight gain and centripetal obesity, hypokalemia) and CS subtype. Patients were then divided in short or long exposure of cortisol excess using the median time of the duration of the disease as cut-off. For the post-operative follow-up analysis, patients with supra-physiological doses of glucocorticoid replacement therapy (i.e., > 30 mg hydrocortisone-equivalent per day), incomplete recovery from CS (as outlined by pathological biochemical tests), and/or cytotoxic treatment (e.g. mitotane or chemotherapy) were excluded.

Due to the well-known effects of menstrual cycle and testosterone levels on erythropoiesis and RBC parameters, these possible confounders were also analyzed at the time of diagnosis of CS. According to menstrual cycle, female patients were stratified into four groups: (i) normal menstrual cycle (with and without contraceptives), (ii) oligomenorrhea (defined as a menstrual cycle of either less than 24 days or more than 39 days), (iii) amenorrhea (iv) menopause. Male patients were sorted according to gonadal function at the time of the diagnosis of CS: (i) age-specific normal levels of gonadotropins and total testosterone, (ii) low levels of gonadotropins and total testosterone [13].

All patients provided written informed consent to at least one of two disease-specific clinical registries (European Network for the Study of Adrenal Tumors, ENSAT, registry and/or Network of Excellence for Neuroendocrine Tumors, NeoExNET, Registry). Both registries were approved by the local Ethics Committee of the University Hospital Würzburg (88/11 for the ENSAT registry and 85/12 for the NeoExNET registry).

### Hormonal analysis

The hormonal analyses were performed with commercially available analytical procedures: the Immulite system (Siemens) for plasma ACTH and serum cortisol, a manual luminescence immunoassay (IBL) for the evaluation of salivary cortisol, and a manual radioimmunoassay (Immuntech) for the analysis of 24 h-urinary free cortisol (UFC), as previously reported [2, 14].

### Red blood cells analysis

Analysis of the RBC parameters, including hematocrit (HCT), RBC count, mean corpuscular volume (MCV), hemoglobin (Hb), mean corpuscular Hb (MCH), and mean corpuscular Hb concentration (MCHC), was performed with GenS Beckman (until 2009), Sysmex XE-2100 (from 2009 until 2017), and Sysmex XN-9000 (from 2017 onwards). The analytical systems did not differ significantly in terms of measurement results and reference values.

### Statistical analysis

Categorical variables are expressed as numbers with percentage and were compared with the Chi-square (χ^2^) test. Continuous variables were tested for Gaussian distribution with the Shapiro–Wilk test. Normally distributed data are presented as mean and standard deviation (SD), while not-normally distributed data are shown as median and interquartile range (IQR). Parametric and non-parametric data were analyzed with Student’s T-tests or ANOVA followed by Tukey *post-hoc* test or Mann–Whitney *U* test or Kruskal–Wallis test followed by Dunn’s *post-hoc* test, respectively, and reported as mean ± standard deviation. Correlation (r) between continuous variables was determined by Pearson’s or Spearman´s correlation coefficient for normally or not normally distributed variables, respectively. For the multivariate regression analysis, the Bonferroni correction was used. To identify the change in RBC parameters after biochemical remission, mean delta change (evaluated in percentage) from baseline was calculated. For this analysis, only individuals with RBC parameters available at baseline and at follow-up were included. A *p*-value < 0.05 was considered statistically significant. Statistical analysis was performed with SPSS version 26 (IBM Corporation, Armonk, NY, USA) and GraphPad Prism version 8 (GraphPad Software, San Diego, CA, USA).

## Results

### Characteristics of the study population

218 patients with endogenous CS were identified. Among these, 8 patients (including 7 women; 5 with CD and each 1 with ECS, CPA, and ACC) were excluded because of supplementation with vitamin B12 (n = 3), iron (n = 2), folate (n = 1) or biochemical evidence of overt hypo- or hyperthyroidism (n = 2) at the time of the initial diagnosis. Hence, the final study population comprised 210 patients [CD, n = 85 (40%); ECS, n = 31 (15%); CPA, n = 46 (22%); ACC, n = 48 (23%)]. Clinical characteristics of the study population are summarized in Table 1.

**Table 1:** Clinical characteristics of the study population with Cushing syndrome at baseline

	Women (n = 162)	Men (n = 48)	p-value
General characteristics			
Age at initial diagnosis of CS (years) [mean, (SD)]	49 (15)	48 (13)	n.s
BMI (kg/m^2^) [median, (IQR)]	29.4 (9.5)	28.7 (5.8)	0.037
Subtype of Cushing syndrome			
Cushing´s disease (%)	66 (41%)	19 (40%)	n.s
Ectopic Cushing´s syndrome (%)	20 (12%)	11 (23%)	n.s
Cortisol-producing adrenal adenoma (%)	40 (25%)	6 (12%)	n.s
Adrenocortical carcinoma (%)	36 (22%)	12 (25%)	n.s
Biochemical analysis			
Serum cortisol after 1-mg DST (µg/dl) [median, (IQR)]	17.4 (14.0)	19.3 (17.8)	n.s
24 h-urinary free cortisol (µg/d) [median, (IQR)]	255.7 (394.4)	297.5 (595.3)	n.s
Late-night salivary cortisol (µg/dl) [median, (IQR)]	0.6 (0.7)	0.9 (2.0)	n.s

ACTH, adrenocorticotropic hormone; BMI, body mass index; DST, dexamethasone suppression test; IQR, inter quartile range; n.s., *p* value not statistically significant; SD, standard deviation

These 210 patients were matched according to sex and age with 117 patients with hormonally inactive pituitary adenomas (without hormonally deficiency) and 93 hormonally inactive adrenal incidentalomas. Median BMI was similar between CS patients and controls (29.0 vs 28.0 kg/m^2^, p = 0.18).

### Characteristics of the female population with Cushing syndrome

The female population with CS included 162 patients (CD, n = 66 (41%); ECS, n = 20 (12%); CPA, n = 40 (25%); ACC, n = 36 (22%)).

**Fig. 1:**
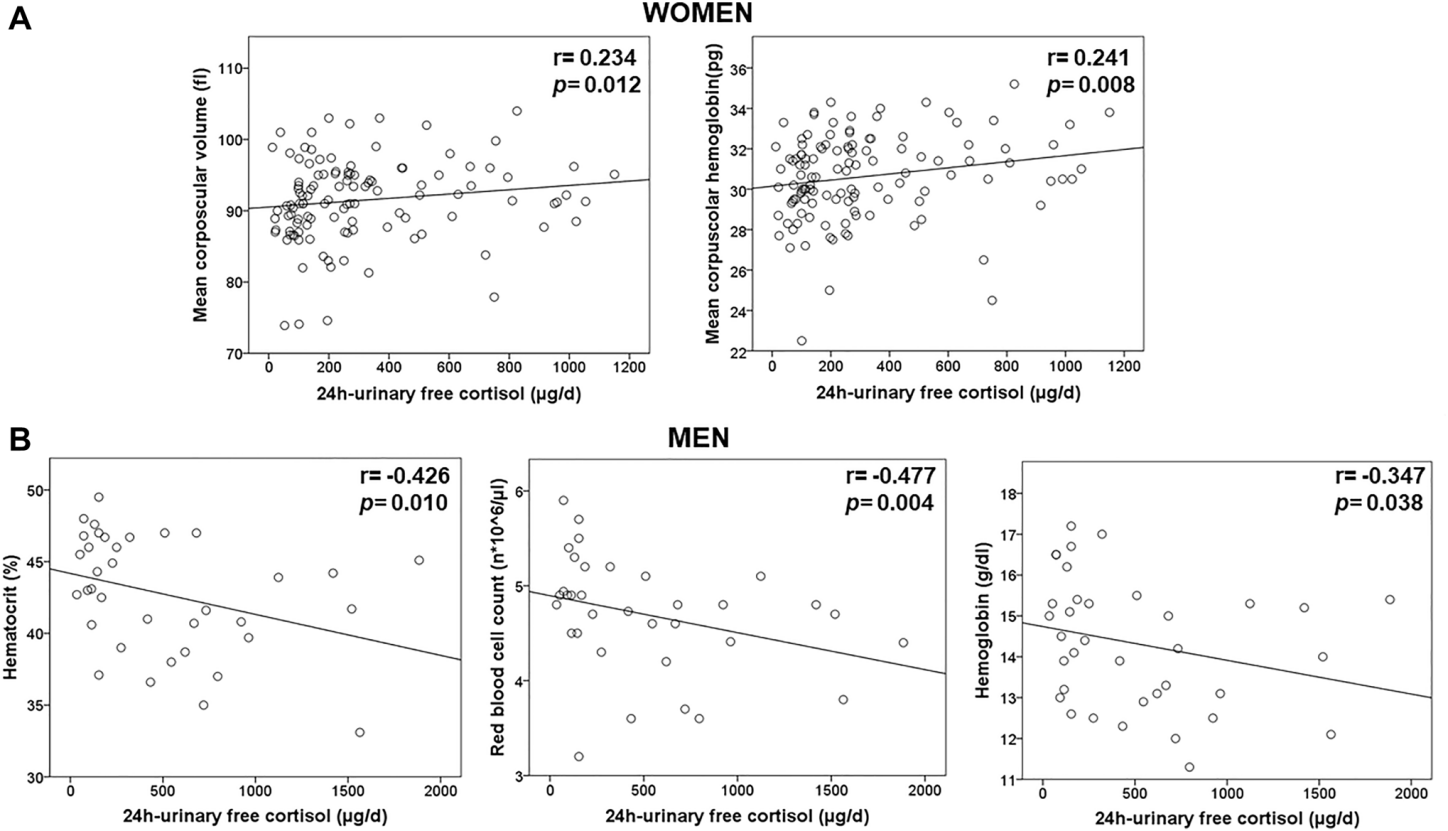
Correlation between 24-urinary free cortisol levels and red blood cells parameters in females (**A**) and males (**B**) at the time of diagnosis of Cushing syndrome. **A** Significant positive correlation between 24-urinary free cortisol levels and mean corpuscular volume as well as mean corpuscular hemoglobin in females with Cushing syndrome. **B** Significant negative correlation between 24-urinary free cortisol levels and hematocrit, red blood cells count, and hemoglobin in males with Cushing syndrome. Correlation (r) was determined by Spearman´s correlation. A *p*-value < 0.05 was considered statistically significant

A significant, but weak positive correlation between both 24 h-UFC and serum cortisol after 1 mg- DST with MCV (24 h-UFC: r = 0.234, 1 mg-DST: 0.229 both *p* < 0.05) and MCH (24 h-UFC: r = 0.241, 1-DST: 0.236, both *p* < 0.05) was identified (Fig. 1.A). No further correlations between 24 h-UFC, serum cortisol after 1 mg-DST, and other RBC parameters were found.

The menstrual cycle status at the time of blood sampling was known in 148 of 162 female patients (91%). 57 patients reported a normal menstrual cycle or took contraceptive at that time; the reminders reported oligomenorrhea (n = 26) and amenorrhea (n = 9) or were in the menopause (n = 56). As shown in Supplemental Fig. 1A, postmenopausal women had significant lower RBC counts compared to those with normal menstrual cycle, oligomenorrhea, and amenorrhea (median 4.5 *vs* 4.8 *vs* 4.9 *vs* 4.8 n*10^6^/µl, all p < 0.05). No differences were observed in the other evaluated parameters (Supplemental Fig. 1A).

Women with CS were matched with 162 women of the control group. CS patients showed higher HCT (median 42.2 *vs* 39.7%,* p* < 0.0001), Hb (14.1 *vs* 13.4 g/dl, *p* < 0.0001), MCV (91.2 *vs* 87.9 fl, *p* < 0.0001), MCH (30.6 *vs* 29.8 pg,* p* < 0.0001) compared to controls (Table 2).

**Table 2:** Comparison between patients with endogenous Cushing syndrome and controls (matched by sex and age)

	Women			Men		
	CS patients (n = 162)	Controls (n = 162)	*p*-value	CS patients (n = 48)	Controls (n = 48)	*p*-value
Hematocrit, %	42.2 (5.5)	39.7 (3.7)	< 0.0001	42.9 (7.3)	44.7 (4.1)	0.027
Red blood cell count, n*10^6^/µl	4.6 (0.6)	4.5 (0.5)	n.s	4.8 (0.8)	5.1 (0.5)	0.001
Hemoglobin, g/dl	14.1 (1.7)	13.4 (1.2)	< 0.0001	14.2 (2.6)	15.4 (1.4)	0.001
MCV, fl	91.2 (8.0)	87.9 (5.1)	< 0.0001	90.8 (6.5)	87.5 (5.4)	< 0.0001
MCH, pg	30.6 (2.8)	29.8 (2.0)	< 0.0001	31.1 (3.2)	30.0 (2.0)	0.020
MCHC, g/dl	33.5 (1.3)	33.9 (1.1)	n.s	33.6 (1.8)	34.6 (1.0)	< 0.0001

Controls were derived from a patient population with both non-secreting pituitary microadenomas or adrenal incidentalomas. Data are shown as median (interquartile range)

CS, Cushing syndrome; IQR, interquartile range; MCH, mean corpuscular hemoglobin; MCHC, mean corpuscular hemoglobin concentration; MCV, mean corpuscular volume; n.s., *p* value not statistically significant

After exclusion of patients with ACC and aggressive neuroendocrine neoplasms, 115 female patients with CS remained. In this subgroup, we observed the same differences to the control group as above mentioned, with the exception of RBC count, which was higher in CS than in controls (Table 3).

**Table 3:** Case–control analysis between patients with endogenous Cushing syndrome without ACC and aggressive neuroendocrine neoplasms, and those with both non-secreting pituitary microadenomas and adrenal incidentalomas (matched by sex and age)

	Women			Men		
	CS patients (n = 115)	Controls (n = 115)	*p*-value	CS patients (n = 32)	Controls (n = 32)	*p*-value
Hematocrit, %	43.0 (4.2)	39.7 (3.5)	< 0.0001	44.1 (5.8)	45.3 (4.0)	0.007
Red blood cell count, n*10^6^/µl	4.7 (0.5)	4.5 (0.5)	0.008	4.8 (0.5)	5.1 (0.4)	0.001
Hemoglobin, g/dl	14.3 (1.5)	13.4 (1.3)	< 0.0001	14.9 (2.3)	15.6 (1.0)	< 0.0001
MCV, fl	92.1 (7.8)	87.5 (5.0)	< 0.0001	89.8 (6.2)	87.6 (5.6)	0.004
MCH, pg	30.7 (2.6)	29.7 (2.0)	< 0.0001	30.4 (3.2)	30.0 (1.0)	n.s
MCHC, g/dl	33.5 (1.3)	34.0 (1.2)	0.055	33.6 (1.8)	34.0 (1.0)	0.008

Data are shown as median (interquartile range)

Controls were derived from a patient population with both non-secreting pituitary microadenomas or adrenal incidentalomas. Data are shown as median (interquartile range)

CS, Cushing syndrome; MCH, mean corpuscular hemoglobin; MCHC, mean corpuscular hemoglobin concentration; MCV, mean corpuscular volume; n.s., *p* value not statistically significant

Slightly more CS patients than controls were under anticoagulants (12 vs 5, *p* = 0.13) and under antiplatelets drugs (12 vs 7, *p* = 0.34).

Twenty-four CS patients experienced a major cardiovascular event within 6 months from diagnosis (Supplemental Table 1). Among these, 2 (8.3%) women had a supraphysiological HCT at diagnosis of CS, both suffering from ACC and deep vein thrombosis (Supplemental Table 1).

To identify if there was a change in RBC parameters related to the duration of hypercortisolism, the interval from appearance of typical CS symptoms until diagnosis of CS was considered. Information about the time of emerging of CS symptoms was available in 94 women. Of those, median time from first symptoms until diagnosis of CS was 12 months. Using the median as a reference, no difference in RBC parameters was observed in patients with a time to diagnosis of maximum 12 months and patients with longer exposure to hypercortisolism (Supplemental Fig. 2A.)

### Characteristics of the male population with Cushing´s syndrome

The male population comprised 48 patients (CD, n = 19 (40%); ECS, n = 11 (23%); CPA, n = 6 (12%); ACC, n = 12 (25%)).

A significant, but weak correlation between 24 h-UFC and HCT (r = -0.426, *p* = 0.01), RBC count (r = -0.477, *p* = 0.004) and Hb (r = -0.347, *p* = 0.038) was identified (Fig. 1.B). Results of the 1 mg-DST were available in 33 out of 48 patients (69%) and did nor correlate with any of the RBC parameters.

Testosterone levels were available in 34 patients (71%). Of note, none of the patients was under testosterone replacement therapy at the time of the RBC count. 14 patients had a normal age-adjusted testosterone level at baseline, while 20 had a biochemical evidence of secondary hypogonadism. The latter patients had significantly lower HCT (median 40.8 *vs* 44.2%, *p* = 0.036) and Hb (13.1 vs 15.0 g/dl, *p* = 0.047) levels compared to those with normal testosterone (Supplemental Fig. 1B). Considering these results and because of the already reported effects of testosterone on RBC parameters [15, 16], a multivariate regression analysis evaluating the impact of 24 h-UFC and testosterone on RBC parameters was performed. The multivariate regression confirmed a significant impact of 24 h-UFC and testosterone on HCT (r = 0.565, *p* = 0.012) and Hb (r = 0.488, *p* = 0.05), while a tendency was observed for the RBC count (r = 0.480, *p* = 0.06).

Compared to the control group, male CS patients showed significant lower levels of HCT (median 42.9 *vs* 44.7%, *p* = 0.027), RBC count (4.8 *vs* 5.1 n*10^6^/µl, *p* = 0.001), Hb (14.2 *vs* 15.4 g/dl, *p* = 0.001), and MCHC (33.6 *vs* 34.6 g/dl, *p* < 0.0001), but higher levels of MCV (90.8 *vs* 87.5 fl, *p* < 0.0001) and MCH (31.1 vs 30.0, *p* = 0.020 pg) (Table 2). Comparable alterations were identified if ACC and aggressive neuroendocrine neoplasms were excluded. The only exception were the levels of MCH, which were similar in patients and control groups (Table 3).

Slightly more CS patients than controls were under anticoagulants (5 vs 2, *p* = 0.43) and under antiplatelets drugs (7 vs 5, *p* = 0.76). Major cardiovascular events were observed in 9 CS patients within 6 months from initial diagnosis. Of note, none of them had a supraphysiological HCT at CS diagnosis (Supplemental Table 1). Twenty out of 48 patients (42%) with CS (5 CD, 5 ECS, 1 CPA, 9 ACC) had Hb levels lower than the age-adjusted normal range. Excluding ACC and aggressive neuroendocrine neoplasms, 11 of 32 patients (34%) had low Hb levels.

Information about the duration of cortisol excess before the diagnosis of CS was available in 33 men. As for the women, median time from first symptoms until diagnosis of CS was 12 months. Also for the men with CS, no difference in terms of RBC parameters according to the short and long exposure of hypercortisolism was observed (Supplemental Fig. 2B).

### Cushing syndrome subtype specific analysis

#### Cushing syndrome subtype specific analysis in the female population

**Fig. 2:**
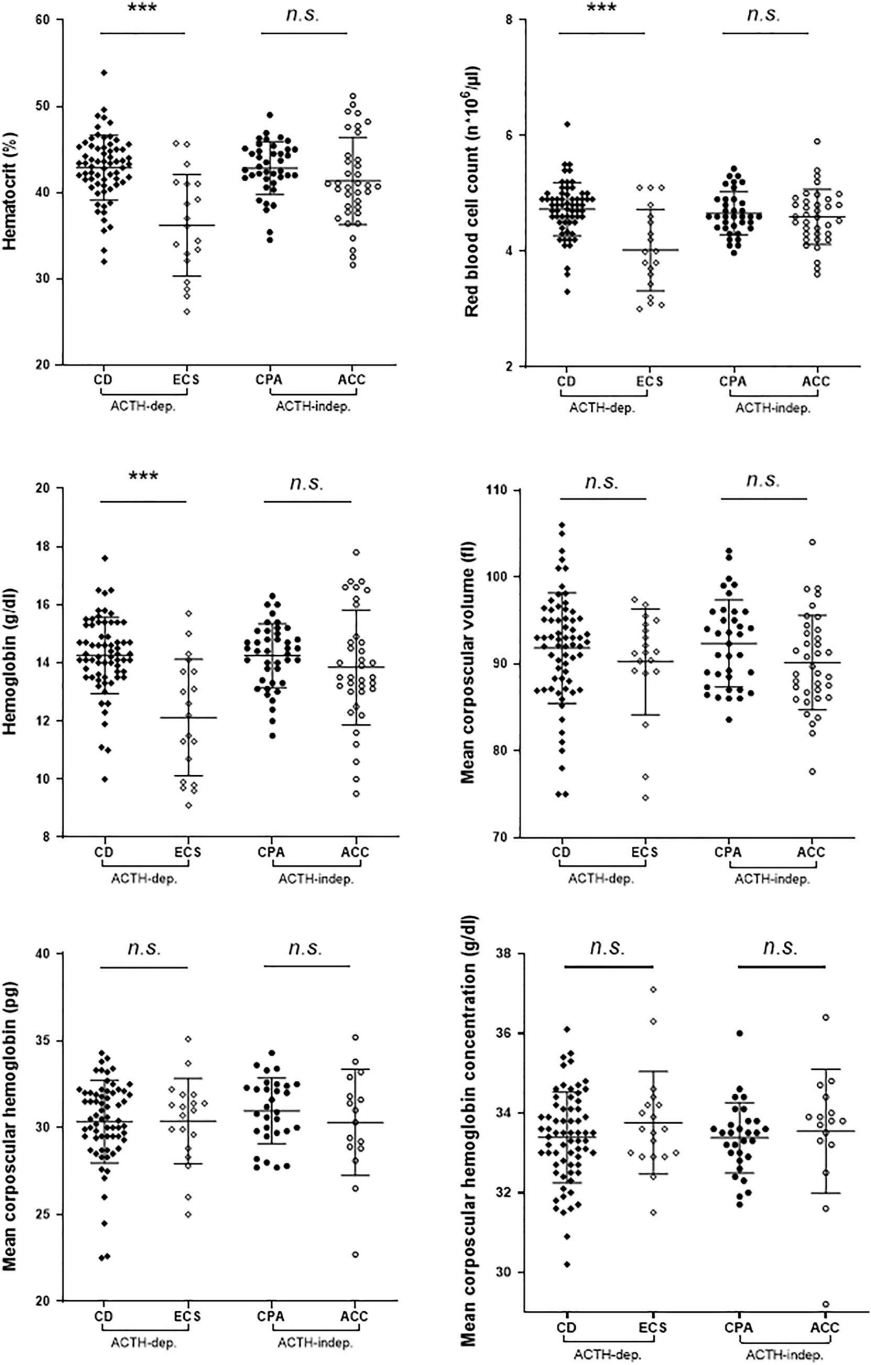
Evaluation of red blood cells parameter in females according to the different subtypes of Cushing syndrome. Among the adrenocorticotropic (ACTH)-dependent (ACTH-dep.) Cushing’s syndrome (CS) subtypes, female patients with Cushing disease (CD) presented significantly higher hematocrit, red blood cell count, and hemoglobin compared to those with ectopic Cushing syndrome (ECS). No difference was identified for mean corpuscular volume, mean corpuscular hemoglobin, and mean corpuscular hemoglobin concentration. The ACTH-independent (ACTH-indep.) CS subtypes (including cortisol producing adenoma, CPA, and adrenocortical carcinoma, ACC) did not differ in any RBC parameters. *p* value format: ***p < 0.001; n.s., *p* not statistically significant

The RBC parameters were analyzed not only according to sex, but also according to the different CS subtypes (Fig. 2). Among female patients with ACTH-dependent CS, those with CD had higher HCT (median 43.3 *vs* 36.1%), RBC count (4.8 *vs* 4.0 n*10^6^/µl), and Hb (14.3 *vs* 12.2 g/dl) than those with ECS (*p* < 0.0005 in all cases). No statistical difference between CD and ECS was identified for MCV, MCH, and MCHC. The ACTH-independent CS subtypes did not differ in any of the RBC parameter. Considering only the 115 patients without ACC and aggressive neuroendocrine neoplasms (66 CD, 9 ECS, 40 CPA), HCT (38.7%), Hb (13 g/dl), and RBC (4.2 n*10^6^/µl) increased in ECS, but all three were still significantly lower than in their CD counterparty (always *p* < 0.005).

The mean and reference values of RBC parameters (according to the mean age of each CS subtype) for the RBC parameters is reported in Supplemental Table 2. The number of CS women with pathological RBC parameters, divided by CS subtype, is reported in the Supplemental Table 3.

#### Cushing syndrome subtype specific analysis in the male population

**Fig. 3:**
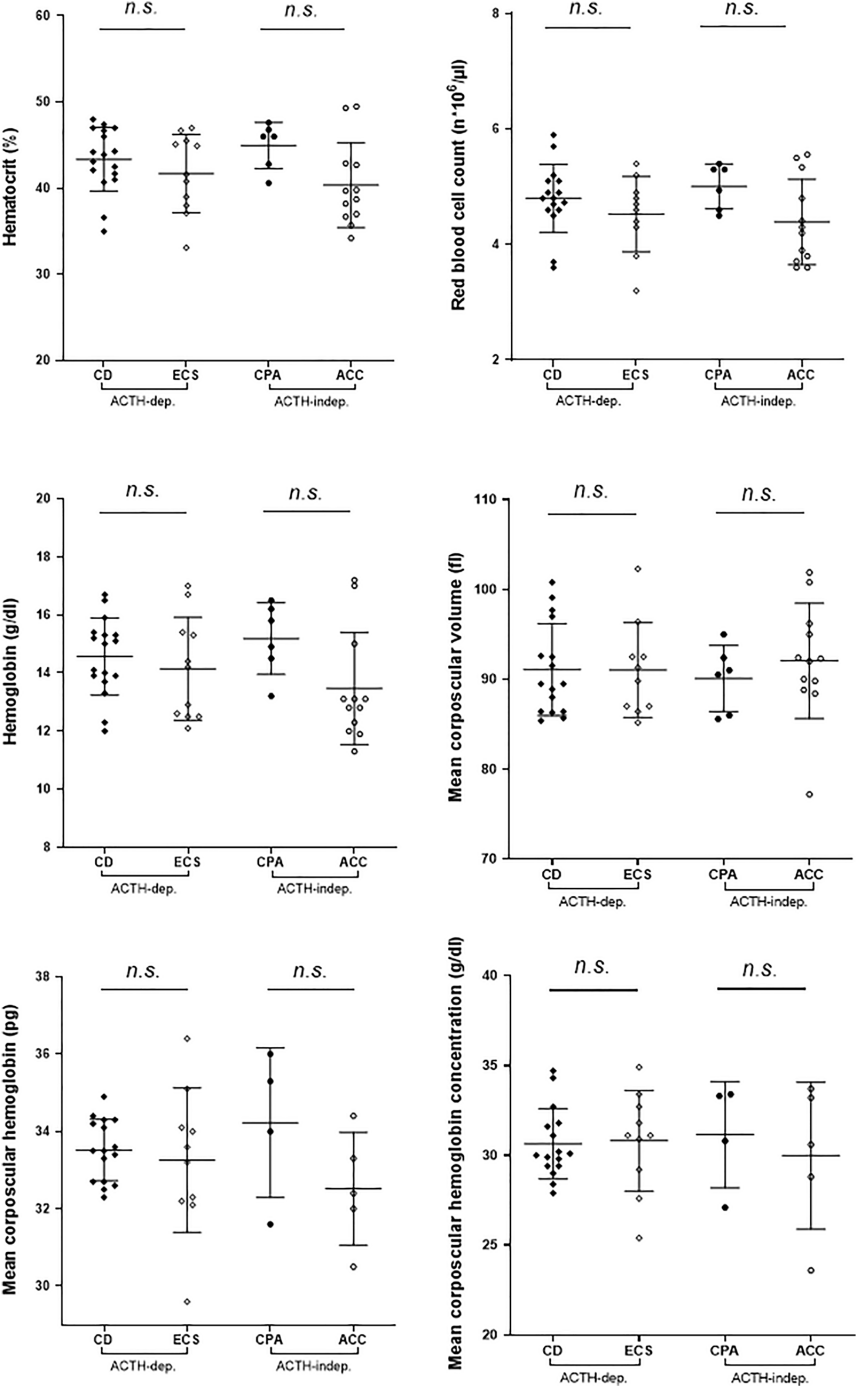
Evaluation of red blood cells parameter in males according to the different subtype of Cushing syndrome. No significant differences between the adrenocorticotropic (ACTH) dependent (ACTH-dep.) and ACTH-independent (ACTH-indep.) Cushing subtypes were observed among the evaluated parameters. Abbreviation: ACC, adrenocortical carcinoma; CPA, cortisol producing adenoma; n.s., *p* not statistically significant

Both the analysis of the different RBC parameters in ACTH-dependent and ACTH-independent CS subtypes (Fig. 3) and the analysis of the 32 CS patients without ACC and aggressive neuroendocrine neoplasms (19 CD, 7 ECS and 6 CPA) did not reveal any significant difference among the groups.

Compared to women with CS, men with CS had a higher proportion of pathological levels for HCT, RBC count and Hb (Supplemental Table 3).

### Changes in red blood cells parameters after remission from Cushing syndrome

#### Red blood cells parameters changes in the female population after remission from Cushing syndrome

24 patients were excluded from the follow-up analysis due to supplementation with iron, vitamin B12 or folate after surgery. Follow-up data at 3, 12, and ≥ 24 months after remission were available from 75, 63, and 52 female patients with CS in remission.

**Fig. 4:**
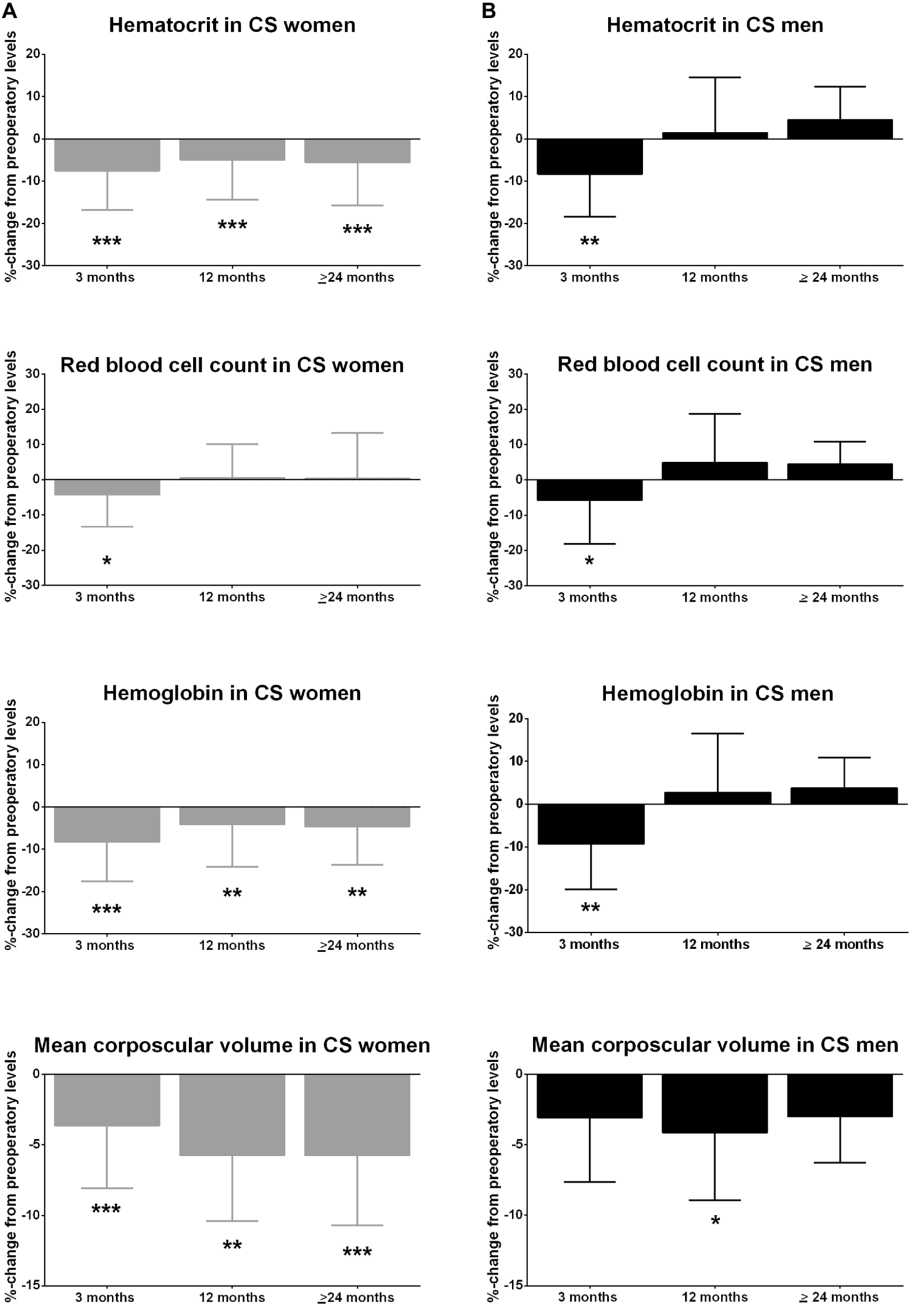
Changes in red blood cells parameters in patients with Cushing syndrome after remission in **A** women and **B** men. Changes in red blood cells parameters were calculated as mean delta change (in percentage) from baseline after 3, 12 and ≥ 24 months from remission of Cushing syndrome. **A** In women with CS in remission, a decrease in hematocrit (HCT), hemoglobin (Hb), and mean corpuscular volume (MCV) was observed over time, with a more pronounced decrease of HCT and Hb during the first three months from remission, and a more pronounced decrease of MCV in the long-term follow-up. After a first decrease of red blood cells (RBC) count at 3 months from remission, an increase was observed over time, reaching levels similar to baseline. **B** In men, a decrease in HCT, Hb, RBC count and MCV was identified at 3 months from CS remission. Over time, HCT, Hb, and RBC increased over the time (at 12 and at 24 or more months), whereas MCV decreased at every time point. Differences of each time point (3, 12, and ≥ 24 months) with baseline were calculated using the Mann Whitney *U* test in both women (**A**) and men (**B**) and indicated as followed if significantly different: *p < 0.05, **p < 0.005, ***p < 0.0001.

At 3 months after surgery, a decrease in HCT (-7.5%), RBC count (-4.3%), Hb (-8.3%), and MCV (-3.7%) was identified (Fig. 4.A), and this pattern persisted until the last follow-up (Fig. 3.A). While the change in HCT and Hb was more pronounced during the first three months from remission, MCV levels progressively decreased over time (− 3.7%, − 5.7%, and − 5.8% at 3, 12 and ≥ 24 months, respectively). On the contrary, after a first decrease of RBC count at 3 months from remission, it increased again up to reach levels similar to baseline (+ 0.5% at 12 months and + 0.4% at ≥ 24 months compared to baseline).

During follow-up no differences in terms of RBC parameters were observed when the different surgical treatments (e.g. pituitary adenomectomy, pulmonary lobectomy, adrenalectomy) were compared.

#### Red blood cells parameters changes in the male population after remission from Cushing syndrome

For the follow-up analyses 2 patients were excluded due to intake of vitamin B12 and due to newly diagnosed hypothyroidism. Accordingly, follow-up data at 3, 12, and ≥ 24 months were available for 23, 17, and 14 male patients with CS in remission.

HCT, RBC count, Hb, and MCV decreased in the first 3 months from CS remission (− 8.3%, − 5.8%, − 9.3% and − 3.1%, respectively, Fig. 4.B). Except for MCV, which decreased at every time point, all parameters increased by the end of the follow-up. Particularly, the increase of HCT was more pronounced at ≥ 24 months (+ 4.5% from baseline), whereas the increase of RBC count (+ 4.9% at 12 months and + 4.5% at ≥ 24 months) and Hb (+ 2.7% at 12 months and + 3.8% at ≥ 24) was constant over the time. As for females with CS, there were no statistical differences in terms RBC parameters related to any surgical treatment.

## Discussion

Our large and comprehensive monocentric study on the association of glucocorticoid excess and RBC in 210 patients with CS shed light on a topic that was largely neglected in the past and, due to conflicting data, not well understood. We describe a relevant sexual dimorphism characterized by an increase in HCT and Hb in women with CS, whereas men with CS showed lower HCT and Hb levels. Furthermore, we performed for the first time a CS subtype specific analysis of the erythropoiesis and identified significant differences in RBC parameters between the ACTH-dependent forms of CS.

Considering that sex-specific discrepancies are increasingly reported in the context of hypercortisolism and CS [7, 17–20] and that CS subtype specific changes in RBC are unknown, the primary aims of this study were to analyze changes in RBC parameters in an heterogenous cohort of CS patients, particularly focusing on sex- and CS subtype-specific differences.

Generally, glucocorticoids are known to stimulate erythropoiesis, thereby increasing the number of RBC [3, 5, 21]. Concordantly, a recent analysis performed on 13 patients with CD revealed an increase in HCT due to cortisol-induced erythrocytosis [5]. Nevertheless, the largest study on erythropoiesis and CS (where only patients with CD were taken into account) did not report an increase in Hb and HCT [7].

In our current analysis, an increase in HCT was identified only in women with overt CS. These results are in line with previous data [5] and explain why glucocorticoids represent a therapeutic option in certain types of anemia [22, 23]. Considering that high HCT is associated to an elevated risk of cardiovascular events [24], also typical of CS [1], we analyzed the HCT of patients with history of cardiovascular episodes. Despite being higher than in controls, the median HCT levels measured in CS women did not exceed the normal range, questioning the contribution of HCT to major cardiovascular events in this patient population. Accordingly, only 2 women with history of cardiovascular events had supraphysiological HCT levels (both slightly over 48%). These two patients had a malignant adrenocortical tumor, which could per se increase the risk of venous thromboembolism [25, 26]. Moreover, none of the male CS patients with a positive history of cardiovascular events had supraphysiological HCT levels at CS diagnosis.

We also observed that females with ECS had lower HCT, RBC, and Hb than those with CD. One explanation might be the presence of malignant disorders in the ECS population. For instance, 5 out of 18 ECS patients had Hb levels below 12 g/dl along with an ectopic ACTH-secretion due to aggressive malignant neuroendocrine neoplasm (2 small cell lung cancers, 2 high grade neuroendocrine neoplasms of the pancreas and 1 atypical pulmonary carcinoid); all of them died within three years from the initial diagnosis of CS. Similarly, 5 ACC patients with Hb levels below 12 g/dl had metastatic disease already at the time of initial diagnosis and died within the first 2 years. This is in line with former publications where lower HCT and Hb in neuroendocrine neoplasms [27] and anemia in cancer patients [28, 29] were described as predictors of poor prognosis.

Nevertheless, if the aggressive neuroendocrine neoplasms were excluded from the analysis, “non-aggressive ECS” still showed significant lower levels of HCT, RBC and Hb than the CD patients. Non-aggressive neuroendocrine neoplasms have already been associated with anemia [30, 31]. Although the idea that RBC analysis may allow for a first subtype differentiation in cases with ACTH-dependent CS subtypes is intriguing, this finding has to be confirmed in larger populations.

An increase in MCV was identified both in females and males with CS. To date, no data on the trophic effect of cortisol on RBC is available. One scenario may be related to a glucocorticoid-induced asymptomatic inflammation of the gastric mucosa followed by malabsorption of vitamin B12 and folate and consequent macrocytosis. In our series, however, vitamin B12 and folate was analyzed in a small subgroup of patients (n = 12), and all of them demonstrated normal values. Moreover, a series of 20 CS patients undergoing gastrointestinal endoscopy did not reveal an increased number of peptic ulcer [32]. Besides, many CS patients suffer from hepatic steatosis [33], and macrocytosis has been observed in this setting [34]. However, a final explanation for the increased MCV levels in CS patients cannot be provided yet.

We identified that males with CS had lower levels of HCT, RBC, and Hb than the control group, thereby confirming the results of a previous study on CD patients only [7]. In contrast, this observation was not made in females. We also observed that reduced RBC parameters in CS male patients were associated with low testosterone levels. As testosterone stimulates erythropoietin and directly acts on the hematopoietic stem cells [15, 35], anemia is a common feature of male hypogonadism [16]. Our results suggest that the gonadal status in men could have a major influence on hematologic function than hypercortisolism. Moreover, considering that hypogonadotropic hypogonadism affects 50–75% of males with CS [1], the risk of anemia should therefore be carefully evaluated in men with CS.

Another relevant aspect is that HCT, Hb and RBC worsened in men after the first months from remission. Of note, although different type of surgeries were performed, in none of the cases was reported a major intra/postoperative bleeding. At least in some patients, the Hb decreased by up to 20%. Accordingly, it is crucial to consider the risk of anemia due to hypogonadotropic hypogonadism, particularly in the first months after curative surgery for CS. Moreover, some of the typical post-remission symptoms that are experienced by CS patients (like fatigue and low quality of life [36]) might be potentially improved by normalizing testosterone levels. On the other hand, it is important to consider that testosterone replacement therapy, if not correctly administrated, can increase the already high risk of thrombosis in CS patients. Furthermore, both HCT, RBC, and Hb on the one hand, and testosterone levels on the other, usually recover over time (as already reported elsewhere [37]).

Some important limitations of our study have to be acknowledged, e.g. its retrospective nature, the small number of patients in whom iron status, vitamin B12, folate have been analyzed (n = 22 (10%), all of them with normal levels), and the large proportion of patients with insufficient data during follow-up. Moreover, although the menstrual state of the women was known in 91% of the case, the exact day of the menstrual cycle at the time of RBC analysis was not available. Furthermore, considering that our center is a referral center for adrenal tumors, the prevalence of CS due to ACC is overestimated compared to other Cushing’s subtypes. Nevertheless, our single center study includes a large cohort of patients with well-defined subtypes of endogenous CS and a homogenous approach to diagnostic workup, treatment, and data collection. Additionally, to avoid the problem and the bias of the high prevalence of ACC, an analysis without CS cancer patients was performed.

In conclusion, we here illustrate how RBC parameters are influenced by endogenous glucocorticoid excess, sex, and CS subtype (particularly in females). The most important alterations in women are an increase in HCT and Hb, independently from gonadal status, whereas men show a decrease in HCT, Hb and RBC count, mostly related to the additional effects of concomitant hypogonadism. This indirectly shows a stronger effect of testosterone than cortisol on erythropoiesis. While increased HCT levels do not result in more major cardiovascular events in women, the risk of anemia in men needs to be carefully evaluated, especially during the first months after remission.

### Supplementary Information

Below is the link to the electronic supplementary material.

Supplemental Figure 1. Red blood cells parameters in patients with Cushing according to (A) menstrual cycle in women and (B) testosterone levels in men. A) Women with menopause (n=56) had significant lower red blood cells count compared to those with normal cycle or under contraceptive therapy (n=57), oligomenorrhea (n=26) and amenorrhea (n=9). . No changes were observed in hematocrit, hemoglobin and mean corpuscular volume in women. B) Men with hypogonadism (n=20) had significantly lower hematocrit and hemoglobin levels compared to those with normal testosterone levels (n=14). No changes were observed in red blod cell count and mean corpuscular volume. Statistical analysis was performed by Kruskal–Wallis test followed by Dunn’s post-hoc test in women (A) and Mann Whitney U test in men (B). p value format: *, p<0.05, ** = p <0.005. Abbreviations: amenor., amenorrhea; contracept., contraceptive therapy; oligomen, olimenorrhea.

Supplemental Figure 2. Analysis of red blood cell parameters according to the duration of Cushing syndrome (from appearance of signs and symptoms, till diagnosis) in women (A) and men (B). A) In women with Cushing syndrome (CS) there was no statistical difference in terms of hematocrit (median 42.70 vs 42.80%, p=0.90), RBC count (4.63 vs 4.60 n*106/µl, p=0.88) hemoglobin (14.10 vs 14.20 g/dl, p=0.92) and MCV (92.3 vs 91 fl, p=0.55) between patients with short time of exposure to cortisol excess (≤12 months) compared to those with long time of exposure (>12 months). B) No difference according to the duration of CS was observed in men (hematocrit (45.1 vs 41.85%, p=0.29), RBC count (4.8 vs 4.6 n*106/µl, p=0.46) hemoglobin (14.5 vs 13.6 g/dl p=0.39) and MCV (91.3 vs 91 fl, p=0.73). Statistical analysis was performed by Mann Whitney U test.

Supplementary file3 (DOCX 14 KB)

Supplementary file4 (DOCX 15 KB)

Supplementary file5 (DOCX 15 KB)

## Data Availability

The authors declare that the data supporting the findings of this study are available within the article.
