# Supplementary material for: Erythropoiesis in Cushing syndrome: sex-related and subtype-specific differences. Results from a monocentric study
Source: J Endocrinol Invest. 2023 Jun 14;47(1):101–13. doi: 10.1007/s40618-023-02128-x (PMC10776705; doi:10.1007/s40618-023-02128-x)
Supplement: Supplementary file 3 — Supplementary file3 (DOCX 14 KB) [file 40618_2023_2128_MOESM3_ESM.docx]

**Supplementary Table 1**. Hematocrit levels at initial diagnosis of Cushing syndrome in patients with history of major cardiovascular events within six months from initial diagnosis of CS.

| **Patient** | **Sex** | **Age at CS diagnosis** | **CS subtype** | **Major cardiovascular event** | **Hematocrit (%)** | **Hematocrit range** |
| --- | --- | --- | --- | --- | --- | --- |
| 1 | F | 40 | CD | DVT+PE | 46.5 | normal |
| 2 | F | 31 | CD | DVT | 45.0 | normal |
| 3 | F | 51 | CD | DVT | 44.8 | normal |
| 4 | F | 58 | CD | DVT | 40.1 | normal |
| 5 | F | 75 | CD | MI | 41.2 | normal |
| 6 | F | 64 | CD | MI | 42.0 | normal |
| 7 | F | 38 | CD | Stroke | 46.1 | normal |
| 8 | F | 61 | ECS | DVT + MI | 32.9 | normal |
| 9 | F | 48 | ECS | PE | 28.8 | normal |
| 10 | F | 53 | ECS | Stroke | 45.6 | normal |
| 11 | F | 64 | ECS | MI | 43.3 | normal |
| 12 | F | 66 | CPA | DVT | 41.1 | normal |
| 13 | F | 68 | CPA | DVT | 42.0 | normal |
| 14 | F | 55 | CPA | Stroke | 41.7 | normal |
| 15 | F | 46 | CPA | Stroke | 41.6 | normal |
| 16 | F | 75 | CPA | MI | 40.3 | normal |
| 17 | F | 65 | CPA | MI | 44.5 | normal |
| 18 | F | 53 | ACC | DVT | 49.4 | high |
| 19 | F | 49 | ACC | DVT | 32.5 | normal |
| 20 | F | 51 | ACC | DVT | 49.2 | high |
| 21 | F | 72 | ACC | DVT + PE | 47.0 | normal |
| 22 | F | 57 | ACC | DVT | 40.6 | normal |
| 23 | F | 73 | ACC | PE | 40.8 | normal |
| 24 | F | 50 | ACC | MI | 34.7 | normal |
| 25 | M | 50 | CD | DVT | 44.3 | normal |
| 26 | M | 46 | CD | DVT | 43.9 | normal |
| 27 | M | 63 | CD | MI | 41.0 | normal |
| 28 | M | 66 | CD | MI | 48.0 | normal |
| 29 | M | 67 | CD | MI | 47.4 | normal |
| 30 | M | 64 | CD | Stroke | 36.6 | normal |
| 31 | M | 58 | ECS | DVT+PE | 40.8 | normal |
| 32 | M | 54 | CPA | PE | 46.1 | normal |
| 33 | M | 44 | ACC | DVT | 38.2 | normal |

Abbreviation: ACC, adrenocortical carcinoma; CD, Cushing disease; CPA, cortisol-producing adenoma; DVT, deep vein thrombosis; ECS, ectopic Cushing syndrome; F, female; high, hematocrit higher than normal range; M, male; MI, myocardial infarction; PE, pulmonary embolism.
