# Supplementary material for: Erythropoiesis in Cushing syndrome: sex-related and subtype-specific differences. Results from a monocentric study
Source: J Endocrinol Invest. 2023 Jun 14;47(1):101–13. doi: 10.1007/s40618-023-02128-x (PMC10776705; doi:10.1007/s40618-023-02128-x)
Supplement: Supplementary file 4 — Supplementary file4 (DOCX 15 KB) [file 40618_2023_2128_MOESM4_ESM.docx]

**Supplemental Table 2.** Mean and normal range of RBC parameters according to the age of each CS subtype.

| **WOMEN** | | | | | | | |
| --- | --- | --- | --- | --- | --- | --- | --- |
|  | **Mean age** | **HCT** | **RBC count** | **Hb** | **MCV** | **MCH** | **MCHC** |
| CD | 45 | 43.9  (36-48%) | 4.8  (4.0-5.0 n*10^6^/µl) | 14.5  (12-16 g/dl) | 91.1  (79-93 fl) | 30.6  (27-31 pg) | 33.5  (32-36 g/dl) |
| ECS | 58 | 36.6  (36-48%) | 4.1  (4.0-5.0 n*10^6^/µl) | 12.3  (12-16 g/dl) | 90.5  (79-93 fl) | 30.9  (27-31 pg) | 33.8  (32-36 g/dl) |
| CPA | 47 | 43.9  (36-48%) | 4.7  (4.0-5.0 n*10^6^/µl) | 14.3  (12-16 g/dl) | 92.4  (79-93 fl) | 30.9  (27-31 pg) | 33.4  (32-36 g/dl) |
| ACC | 52 | 41.3  (36-48%) | 4.6  (4.0-5.0 n*10^6^/µl) | 13.8  (12-16 g/dl) | 90.1  (79-93 fl) | 30.4  (27-31 pg) | 33.4  (32-36 g/dl) |
| **MEN** | | | | | | | |
|  | **Mean age** | **HCT** | **RBC count** | **Hb** | **MCV** | **MCH** | **MCHC** |
| CD | 47 | 43.5  (40-54%) | 4.8  (4.5-5.9 n*10^6^/µl) | 14.5  (14-18 g/dl) | 91.1  (79-93 fl) | 30.6  (27-32 pg) | 33.5  (32-36 g/dl) |
| ECS | 48 | 42.1  (40-54%) | 4.5  (4.5-5.9 n*10^6^/µl) | 14.5  (14-18 g/dl) | 90.8  (79-93 fl) | 31.5  (27-31 pg) | 33.3  (32-36 g/dl) |
| CPA | 53 | 45.0  (40-54%) | 5.0  (4.5-5.9 n*10^6^/µl) | 15.2  (14-18 g/dl) | 90.1  (79-93 fl) | 31.1  (27-31 pg). | 34.0  (32-36 g/dl) |
| ACC | 46 | 40.4  (40-54%) | 4.4  (4.5-5.9 n*10^6^/µl) | 13.5  (14-18 g/dl) | 92.1  (79-93 fl) | 31.3  (27-31 pg) | 33.5  (32-36 g/dl) |

In red are reported the values outside the normal range.

Abbreviation: ACC, adrenocortical carcinoma; CD, Cushing disease; CPA, cortisol-producing adenoma; ECS, ectopic Cushing syndrome. Hb, hemoglobin; HCT, hematocrit, MCH, mean corpuscular hemoglobin; MCHC, mean corpuscular hemoglobin concentration; MCV, mean corpuscular volume, RBC count, red blood cell count
