# Supplementary material for: Erythropoiesis in Cushing syndrome: sex-related and subtype-specific differences. Results from a monocentric study
Source: J Endocrinol Invest. 2023 Jun 14;47(1):101–13. doi: 10.1007/s40618-023-02128-x (PMC10776705; doi:10.1007/s40618-023-02128-x)
Supplement: Supplementary file 5 — Supplementary file5 (DOCX 15 KB) [file 40618_2023_2128_MOESM5_ESM.docx]

**Supplemental Table 3.** Number of CS patients with RBC parameters outside the normal range, divided by sex and by subtype.

|  | **Women** | | | | | **Men** | | | | |
| --- | --- | --- | --- | --- | --- | --- | --- | --- | --- | --- |
|  | **CD**  **(tot=66)** | **ECS**  **(tot=20)** | **CPA**  **(tot=40)** | **ACC**  **(tot=36)** | **Total patients with path. results** | **CD**  **(tot=19)** | **ECS**  **(tot=11)** | **CPA**  **(tot=6)** | **ACC**  **(tot=12)** | **Total patients with path. results** |
| Hematocrit outside NL | 12  (10H, 2L) | 9  (9L) | 2  (1H, 1L) | 9  (5H, 4L) | 32  (16H, 6L) | 2  (2L) | 3  (3L) | 1  (1L) | 8  (8L) | 14  (14L) |
| Red blood cell count outside NL | 14  (11H, 3L) | 9  (9L) | 7  (7H) | 5  (2H, 3L) | 35  (20H, 15L) | 3  (3L) | 2  (2L) | 2  (2L) | 8  (8L) | 15  (15L) |
| Hemoglobin outside NL | 5  (2H, 3L) | 9  (9L) | 4  (3H, 1L) | 12  (7H, 5L) | 30  (12H,18L) | 7  (7L) | 3  (3L) | 1  (1L) | 9  (9L) | 20  (20L) |
| MCV outside NL | 19  (15H, 4L) | 4  (2H, 2L) | 9  (9H) | 6  (5 H, 1L) | 38  (31H, 7L) | 4  (4H) | 1  (1H) | 0 | 3  (2H, 1L) | 8  (7H, 1L) |
| MCH outside NL | 33  (30H, 3L) | 12  (10H, 2L) | 19  (19H) | 15  (13H, 2L) | 79  (72H, 7L) | 7  (4H, 3L) | 8  (7H, 1L) | 3  (3H) | 9  (8H, 1L) | 27  (22H, 5L) |
| MCHC outside NL | 3  (2H, 1L) | 2  (2H) | 1  (1H) | 2  (1H, 1L) | 8  (6H, 2L) | 0 | 2  (2H) | 0 | 0 | 2  (2H) |

Abbreviation: ACC, adrenocortical carcinoma; CD, Cushing disease; CPA, cortisol-producing adenoma; ECS, ectopic Cushing syndrome; H: number of patients with levels above the normal limit; Hb, hemoglobin; HCT, hematocrit; L: number of patients with levels below the normal limit; MCH, mean corpuscular hemoglobin; MCHC, mean corpuscular hemoglobin concentration; MCV, mean corpuscular volume; NL: normal limit; RBC count, red blood cell count
